# 1655. Factors Associated with Acceptance of Telehealth-based Antimicrobial Stewardship Program Recommendations in a Community Hospital Health System

**DOI:** 10.1093/ofid/ofac492.121

**Published:** 2022-12-15

**Authors:** Nathan R Shively, Max W Jacobs, Matthew A Moffa, Rebecca E Schorr, Thomas L Walsh

**Affiliations:** Allegheny Health Network, Pittsburgh, PA; Allegheny Health Network, Pittsburgh, PA; Allegheny Health Network, Pittsburgh, PA; Highmark Health, Pittsburgh, Pennsylvania; Allegheny Health Network, Pittsburgh, PA

## Abstract

**Background:**

Telehealth-based antimicrobial stewardship programs (TeleASPs) have led to reduced broad-spectrum antimicrobial utilization. Data on factors associated with acceptance of stewardship recommendations are limited.

**Methods:**

A TeleASP, facilitated by remote infectious disease physicians and local pharmacists, was implemented in 2 community hospitals from February 2018 through July 2020. Variables potentially affecting acceptance of TeleASP recommendations were tracked. Odds ratios of acceptance were determined utilizing multiple logistic regression.

**Results:**

During the 30 month period, 4863 (91.2%) of the total 5333 recommendations were accepted. Hospitalist and Critical Care Medicine services had significantly higher odds of acceptance in univariable analysis, while General Surgery, Private Practice Primary Care Physician, Pulmonology, and Urology had lower odds of acceptance. Only Critical Care Medicine remained significant on multivariable analysis (OR 3.23, 95%CI 1.4–7.5). Other factors associated with a higher odds of acceptance in multivariable analysis were recommendations for antimicrobial dose/frequency adjustment (OR 2.63, 95%CI 1.6–4.3) and order for labs/tests (OR 3.30, 95%CI 2.1–5.2), while recommendations for antimicrobial de-escalation (OR 0.75, 95%CI 0.60–0.95) and antimicrobial discontinuation (OR 0.57, 95%CI 0.42–0.76) were associated with lower odds of acceptance. Female physicians were more likely to accept recommendations compared to males (93.1% vs 90.3% acceptance, OR 1.65; 95%CI 1.3–2.2). Compared to physicians with less than 3 years of experience, who had the highest acceptance rate (96.3%), physicians with 21 or more years of experience had the lowest (87.1%, OR 0.26; 95%CI 0.15–0.45).

**Conclusion:**

TeleASP recommendations were accepted at a high rate. Acceptance rates were higher among female physicians, and recommendations to stop or de-escalate antimicrobials led to lower acceptance. Recommendations made to the most experienced physicians were the least accepted, which may be an important factor for stewardship programs to consider in education and intervention efforts.

**Disclosures:**

**Thomas L. Walsh, MD**, Accelerate Diagnostics: Honoraria.

